# Hospital and Institutional News

**Published:** 1919-05-31

**Authors:** 


### HOSPITAL AND INSTITUTIONAL NEWS.

### R.A.M.C. MEMORIAL SERVICE.

THE Memorial Service previously announced to take place in St. Paul's Cathedral at twelve noon on June 12 has, we understand, been unavoidably postponed until June 25, the place and time, however, remaining unchanged. Applications for tickets should be made to Captain A. R. Wright, D.S.O., R.A.M.C., Army Medical Service, War Office, Adastral House, Victoria Embankment, before June 12. Those already received for the earlier date will be noted for the service on the 25th, unless request is made to the contrary.

### THE MOTOR AMBULANCE DINNER.

ON this date a Red Cross motor-ambulance dinner will be held at the Connaught Rooms, Great Queen Street, Holborn, at 6.30. Sir Ernest Clarke, director of the motor-ambulance department of the British Red Cross, will preside, and Sir Arthur Stanley, G.B.E., chairman of the Joint War Committee, will be the guest of the evening. The occasion will be not only a reunion of the ambulance men, but also the occasion on which it is hoped to raise £1,000 to endow a bed to those of the corps who lost their lives in the war. A memorial tablet will be placed in St. James's, Piccadilly, where, at 3 P.M. on the same day, the rector will hold a service.

### BUBONIC PLAGUE AT LIVERPOOL.

A SOLDIER passenger on board the S.S. "City of Sparta," which left Bombay on April 3, was taken ill during the voyage, and died of bubonic plague. He was buried at sea. The vessel, on arrival at Liverpool on April 29, was treated as a suspected ship and diverted to the mooring station, where all the usual precautions were adopted; portions of the ship were fumigated with sulphurous-acid gas, and subsequently sprayed with petroleum emulsion. The Local Government Board have now received an intimation that a native member of the crew fell ill and was removed to the Liverpool Port Sanitary Hospital on May 13. He developed a bubo on May 16, and died next day. The illness was diagnosed as plague, and this diagnosis has been verified by bacteriological examination. The Local Government Board are making further inquiry in regard to this case through one of their medical inspectors.

### HOSPITAL FOR TROPICAL DISEASES.

ANNOUNCEMENT was made in THE HOSPITAL of March 15 last that the British Red Cross Society and Order of St. John had offered to purchase for the Seamen's Hospital Society premises near University College, London, to provide medical and surgical treatment for sailors and soldiers who had contracted tropical diseases while on active service, and, ultimately, when this serious need has been met, for the benefit of sailors. It is now stated that the premises referred to are the Endsleigh Palace Hospital for Officers, formerly the Endsleigh Palace Hotel, Euston Square. The new hospital will contain fifty beds, thirty of which are to be endowed by the Joint War Committee of the British Red Cross Society and the Order of St. John. The London School of Tropical Medicine, which is a branch of the Seamen's Hospital Society, will be attached to the new establishment.

### ADVERTISED PATENT REMEDIES.

IT is satisfactory to note that during the past week the attention of Parliament has been again directed towards the case of alleged fraudulent remedies for the prevention and cure of disease. These matters have been carefully discussed by a Select Committee of the House of Commons on Patent Medicines, and by whatever legislative mechanism the problem is ultimately treated, there is no doubt that a satisfactory solution is urgently necessary. Several times has the subject been mentioned by this journal, and instances where direct or indirect injury has occurred to the public health may be multiplied indefinitely. Previously many cases have gone unpunished because it was nobody's business to initiate prosecution, yet the bad effect must have been obvious to any one however slight his medical knowledge. Perhaps we may now hope soon to see a clean sweep made of these supposed helpers, but actually greatest enemies, of organised preventive medicine.

### MEDICAL INSPECTION OF SCHOOLS.

IN the Paris *Médical* for February 8, Théry remarks that medical inspection of schools dates at Paris from 1879, but no attempt has been made until the last decade to extend the work of the medical inspectors beyond the defence of the school against contagion. In 1910 the city was divided into 210 medical districts for the purpose of school inspection, each inspector having about a thousand children allotted to him. Each child is soon to have its individual health booklet, to record the results of regular examinations through the entire school life, and possibly later. The inspector should record the weight, height, and chest measure, etc., the spirometer findings, the effect of test exertion on the heart, the acceleration of the pulse and arterial tension; also the results of muscular tests with the dynamometer. There should be an obligatory general examination every two years. The inspector should be familiar with the normal standards and the normal development of body and mind, and he should be paid enough so he need not be obliged to regard this school work as merely an accessory to his professional work. The making out of the health booklets and the thorough examination as the child enters school for the first time should be regarded and remunerated as an extra function, aside from the habitual duties of the school physician. His routine duties include prophylaxis of contagious diseases and control of the physical education, and these require an inspection visit to the school once a week, or possibly twice under certain conditions. His task might be singularly facilitated by the creation of school nurses. Such a scheme, once working, would undoubtedly prove of the greatest national value. We hope, in the coming health reforms, to see attention directed to practical as well as theoretical efficiency of school inspection work.

### REPORTS OF MISTAKES.

DR. MARTIN ALVAREZ, in his article on "Blunders" in the March number of the *Bulletin* of the Porto Rico Medical Association, asks his colleagues to report their blunders, deeming this the most instructive way of learning from experience. He thinks the time has come when the study of blunders one has made or that have been made by his colleagues will teach much more than can be learned from the text-books. He remarks that when the young graduate leaves college he always wants to become a surgeon; in the first place because the knife represents "el sport de la muerte," and in the second place because he believes that the "bistoury is a synonym for California." Alvarez wants to start a "Mistakes Section" in the *Bulletin*, and he invites others to aid him, saying that in the next issue he will begin to publish his own mistakes, and adds, "Who will follow me?"

### TO HOSPITAL ARCHITECTS.

THE borough of Islington's war memorial is to be the extension of the Great Northern Central Hospital, the cost of which is expected to be £100,000. The plans will be selected from a competition, for which Sir Aston Webb, F.R.I.B.A., P.R.A., will be the assessor.

### WELL-KNOWN HOSPITAL ARCHITECT DEAD.

MR. S. PERKINS PICK, who died recently at Leicester, was an architect whose knowledge of hospital architecture was the result of long and constant study. His work for the Leicester Royal Infirmary has frequently been reviewed in THE HOSPITAL. Other institutions which have enjoyed his supervision architecturally are the Leicestershire County Asylum, the Leicester Borough Asylum, Addenbrooke's Hospital, Cambridge, the Royal Hants County Hospital, Winchester (recent developments), and the Coppice, Nottingham. Mr. Pick was architect to the Leicester County Council, the Leicester Borough Council, and many local bodies. Recently he was selected by the Council of the R.I.B.A. to go to France and Germany to confer with and advise young officers as to the possibilities of the profession, etc., on their return to private life. It was while on Government duty that the illness developed from which he never recovered.

### THE MEDICAL SCHOOL AT LEEDS.

THE School of Medicine at Leeds University is to be expanded, so that clinical training may be embraced. An arrangement, therefore, has been made with the General Infirmary, and the City Council's Health Committee, whereby the University School of Pathology and Bacteriology may, it is hoped, develop into a properly equipped pathological institute. Neighbouring buildings have been bought for the purpose, and better salaries will be provided and ex-officers trained if only some one will relieve the University of the impecuniosity which now hampers it in every direction.

### THE CARDIFF CHAIR OF PREVENTIVE MEDICINE.

THE Welsh National Medical School at Cardiff has begun to be an educational reality with the appointment of Dr. Edgar Lee Collis, F.R.C.P., of Oxford and St. Thomas's Hospital, as first Professor of Preventive Medicine. The appointment must be associated with the late Miss Talbot, of Margam Abbey, who, shortly before her death, handed a sum yielding £1,500 a year to the trustees, Lord Aberdare, Sir William Osler, and Sir Arthur Way. It was the duty of the trustees to nominate a first professor of preventive medicine and to draft a scheme agreeable to Cardiff College. The electors had chosen Dr. E. L. Collis, who, for the past two years has been Director of Welfare and Health under the Ministry of Munitions. Before that his experience was wide. After leaving Oxford with first-class honours in the School of Natural Science, and completing his medical education at St. Thomas's Hospital, Dr. Collis practised at Stourbridge before becoming for nine years a medical inspector of factories for the Home Office. By his appointment to the chair of Preventive Medicine at Cardiff he becomes the nucleus of the teaching staff. As Colonel Bruce Vaughan has often urged, in the creation of a new medical school, the highest qualifications are essential. The tradition must be unquestionable at its beginning, if a high reputation is to be maintained or even won.

### THE PRIDE OF CARDIFF CITY.

WHEN a growing hospital is forced to make frequent appeals for money, it is a good thing to formulate its growth rather as a succession of triumphs than a long and depressing list of needs. Now, therefore, Colonel Bruce Vaughan, in boldly launching a new appeal for the building fund of King Edward VII.'s Hospital, Cardiff, very wisely puts to the front some of the things which have been done since previous appeals were made. He turns to his public and reminds it of that which it has done already, in order to persuade it to surpass itself. Hardened as we are to appeals, we catch something of his own infection. When he points to the one hundred beds which *have* been added since the last appeal to the Maternity department which has *been* opened, to the Lee Convalescent Home *now* at work, to the 200 further beds which "are more than half finished," we feel the thrill of realisation. It becomes almost a reflex emotion of the will to desire to make the further £35,000 needed another accomplished fact. After all, how small that sum is to such a city, the Babylonian port of Wales? Nor is civic pride forgotten. The hospital is to be, he tells us, beautiful as well as useful, and are not the pictures in its wards already one of the sights of the city? The façade is now to be carved with the symbols and mottoes of all the Welsh Regiments, as well as those of the Royal Flying Corps and the Navy. The only thing which can give to a town either the dignity or repose of the countryside is architecture. What is the use of a city which can boast only of its wealth, if its buildings do not display its magnificence in their architecture? Without that Cardiff would remain a Babylon in everything but beauty.

### ST. LUKE'S HOSPITAL FOR MENTAL DISEASES.

This hospital, which, after 165 years' work in Old Street, E.C., has been closed since December 1916, the land on which it stood being bought by the Bank of England, is to be re-established in more favourable surroundings in the country. For this purpose land has been acquired near Gerrards Cross, Bucks, and it is hoped to begin building as soon as circumstances will permit. Meantime the business affairs of the hospital are conducted from its offices, 19 Nottingham Place, London, W. 1.

### THE NEWEST HOSPITAL FOR WOMEN.

From a humble beginning the new Sussex Hospital for Women and Children at Brighton emerges into the mid-stream of hospital affairs. Its present home cannot be enlarged, so the committee is asking for £50,000 to re-build it. This sum could be raised in five minutes at Brighton, since the air raids drove so many of the rich and timid out of town. It is still packed with wealthy people, but, since these may want something more than a simple need to stir them, they can now be in the fashion by supporting a scheme which offers the services of women doctors to patients of the same sex. Moreover, the new Sussex Hospital has some provision for paying patients, and its fees for this class range from 15s. to 84s. a week. Sir John Havard's executors have enabled a suitable site to be bought, on which a hospital of fifty beds could be built conveniently.

### THE DILEMMA AT DARLINGTON.

The proposal to build a new hospital for Darlington is advanced by the purchase of a property of seventeen acres called "The Elms." Building, however, will be postponed until the capital expenditure is forthcoming. The need for activity can be judged from the fact that existing accommodation is so severely limited that the committee has declined to admit cases of ophthalmia neonatorum for in-patient treatment. If there is no room for babies suffering from a disease which, unless tackled as it cannot be at home, produces blindness, Darlington Hospital is disgracefully inadequate to the town. Is no one public-spirited enough to come to the rescue?

### HELPING INVALID CHILDREN.

At the annual meeting of the Invalid Children's Aid Association, Sir Arthur Stanley, who presided, announced the gift by Mr. Charles Lee of the Red Cross Hospital, Crosland, Stonebridge Park, near Willesden, as a convalescent home. This is the first result of the efforts to utilise the organisation of voluntary work under the Red Cross Society for the care of children throughout the United Kingdom. The extent of the Association's work may be gathered from the following statistics for 1918: Number of cases referred, 7,515; sent away, 10,557; carriages lent, 5,262; surgical instruments (boots and crutches), 1,560; apprenticed and trained, 313; total amount raised, £61,074. There are twenty-two districts working under the Central Committee, and twenty-two branches with sub-committees of their own. The work is increasing, and there is need for more helpers and larger financial support.

### SELF-HELP AT ABERDEEN.

For a number of years the Royal Aberdeen Hospital for Sick Children has been fortunate in having the expenses of its little Convalescent Home for eleven patients largely defrayed by a lady interested in the welfare of children, to whom its management has been a labour of love. This year, however, the hospital becomes responsible for its upkeep, and the staff, keen on open-air treatment, has just raised £184 by a successful concert, for the erection of an open-air pavilion at the home. This, it is hoped, will accommodate twenty children, and leave the home for the use chiefly of the staff. Up to the present the staff has suffered cheerfully under very cramped conditions, which the lady superintendent has long desired to alter.

### THE HABIT OF MAGNIFICENCE.

It is interesting to note that Mr. D. Mason, whose gift of £22,000 to the West London Hospital has given a good start to its appeal, does not confine his public spirit to hospitals. His firm, whose Cherry Blossom boot polish is familiar alike in use and advertisement, has arranged for Saturday to be a whole holiday for all, or nearly all, of its employees, who have the run of tennis courts and gymnasium and an educational course. If only this public sense were as common in the ranks of commerce as it is among the big and successful firms, trade and men of business would stand higher than they do. For it is one of the first lessons of life that the habit of magnificence is not a matter of income; it is an attribute of character which distinguishes the commoner from the clown. An orgy of expenditure can achieve only vulgarity, for the habit is a matter, not of riches, but of style. Midas in his motor-car is, strictly, a road-hog.

### AN INGENIOUS FUND.

At Downing Street last week a meeting was held in aid of the Ivory Cross National Dental Aid Fund. Founded by Miss Fletcher, the fund is achieving a double purpose, that of enabling a patient anywhere to go to the nearest qualified dentist from whom to receive the same treatment as a paying patient, and that also of giving to dental surgery in this country the recognition which it has been slow to receive. The war taught us how expensive to the national health neglected dental caries was, and the 1,500 or more dentists on the roll of the fund have done something to provide the treatment for which otherwise many thousands would have had to wait in vain. Through the offices at 10 Conduit Street thousands of patients have passed, including last year some 12,000 discharged from the Army, and expectant mothers have also received treatment. The fund, like an institution, has a long waiting-list, but, unlike a hospital, its expenses are relatively small. Its method consists in decentralisation, and its rôle is the equivalent of an organisation of private practice for its patients. Instead of hurrying a man to hospital, it virtually sends the dentist to his own door. It gives the benefits of private practice to the poor, and shews hopefully how much can be done by a voluntary system, on the one hand, and by decentralisation on the other.

### A HOSPITAL FOR EGHAM.

A memorial scheme for a new hospital which appears to be well started is that adopted by the people of Egham. Mr. W. G. Rigden has promised a site, and Mr. Hartley, of Englefield Green, £1,000 to endow a free bed for soldiers and sailors who have fought in the War.

### ROYAL NAVAL HOSPITAL, CHATHAM.

On his retirement after thirty-eight years in the Navy, of which thirteen have been passed at the Royal Naval Hospital, Chatham, Commissioned Wardmaster F. Hannaford has been presented with an illuminated address, and Mrs. Hannaford with a silver tea-service. The presentation is a tribute to his good work for the benefit of the sick berth staff. He gave this parting message, which all married men in hospital life may take to heart: "Where wives take an interest in their husbands' duties, it is for the well-being of the staff in general."

### HOSPITALS ON THE FILM.

The cinematograph, which has already been used for the professional training of undertakers, is being put to a more cheerful use in acquainting its patrons with life in a hospital ward. By arrangement with various "Empires," a film descriptive of "A Visit to the Great Northern Central Hospital" is being exhibited at various places of entertainment in North London, and collections have been taken for the hospital's benefit. Let us say at once that this has none of the objections which attached to the participation of the profits of Sunday cinematograph shows of no hospital interest whatever. No sensible man, on reflection, would wish to hurt the Sunday Fund, as participation in Sunday performances nearly did. But, apart from this capital objection, a hospital, as far as possible, should raise its money by interesting the public *in its own work*, and should dispense, as far as possible, with the shifts of commerce.

### DR JOHNSON ON ADMINISTRATION.

The practice of having a number of ornamental names associated with hospitals is less extensive than it used to be, and gigantic committees are beloved only by the leisured world of labour, which is apt at first to mistake interference for management. But the force which counts in hospital administration was well put by Dr. Johnson, and experience will bear him out. "Though many men," Boswell once heard him remark, "are nominally intrusted with the administration of hospitals and other public institutions, almost all the good is done by one man, by whom the rest are driven on; owing to confidence in him, and indolence in them." Though this is true, human vanity is such that each is apt to regard himself as indispensable, in spite of the fact that the death of one man rarely prevents a well-managed institution from maintaining its prosperity.

### THE AUTHOR TO READ IN HOSPITAL.

The following confession is worth recording: "I have been re-reading," a busy hospital worker writes to us, "a Henry James favourite—'What Maisie Knew'—which I really enjoyed very much. Henry James gives you the feeling that he never had to earn his living, and never ran for a train in his life; so he is the very person to read in hospital, where one is always hurrying." The hospital world is vivid, but there is another world, and one welcomes this appreciation of the contrast. If the busy people knew as much about leisure as Henry James knew about work, the machinery of existence would run more smoothly, and be gayer and more humorous than it is.

### INSTITUTIONAL BEE-KEEPING.

A suggestion is made that bee-keeping might well be a profitable enterprise for hospitals and other institutions, especially those situated in country districts. The ground space required is small and, after the initial outlay, the expenses are trifling. Under good management fresh stocks of bees can be raised annually from the original hive, and each established stock may be expected to yield 60 to 80 lb. of honey in the season. The idea is, perhaps, worthy of consideration.

### A SIGN OF THE TIMES.

The Ladywell Institution was built at enormous expense some years ago by the Bermondsey Board of Guardians for the accommodation of their aged poor. It is a splendid institution in the midst of beautiful grounds, its only drawback being that it is situated a considerable distance from Bermondsey. Soon after war broke out the Army Council converted it into a hospital for wounded soldiers, and now that it is no longer needed for that purpose it is being used as a place for the training of convalescent and disabled soldiers in trades and technical work. The Guardians are desirous that the Army Council shall take over the institution entirely, for their poor have so greatly diminished in number that there is ample accommodation for them elsewhere. This is a sign of the times.

### THIS WEEK'S DRUG MARKET.

Business is rather less active than it was during the past two weeks, but there is still a good deal of inquiry and the tone of the market is fairly healthy. As the Whitsuntide holiday approaches we may expect to see a temporary falling off in business, but we may take it that the improvement will probably be maintained, with a dull period now and then intermingled with an active one. Menthol and camphor continue to attract attention, and both are again dearer. Cardamoms are in good demand and the price tendency is still upward. Calumba root is cheaper with the arrival of larger supplies. Castor oil, linseed, linseed oil, and rape oil are all dearer. Gentian root has an upward price tendency. The improved demand for aniseed oil has not been maintained and the price is rather lower. With arrivals of Turkey opium prices are likely to be much lower in the near future, and quotations for morphine and codeine will probably be reduced.

